# Effectiveness of COVID-19 vaccines against SARS-CoV-2 infection with the Delta (B.1.617.2) variant: second interim results of a living systematic review and meta-analysis, 1 January to 25 August 2021

**DOI:** 10.2807/1560-7917.ES.2021.26.41.2100920

**Published:** 2021-10-14

**Authors:** Thomas Harder, Wiebe Külper-Schiek, Sarah Reda, Marina Treskova-Schwarzbach, Judith Koch, Sabine Vygen-Bonnet, Ole Wichmann

**Affiliations:** 1 Robert Koch Institute, Berlin, Germany

**Keywords:** SARS-CoV-2, systematic review, vaccine effectiveness, vaccination, COVID-19, Delta variant, variant of concern

## Abstract

The Delta variant has become the dominant strain of SARS-CoV-2. We summarised the evidence on COVID-19 vaccine effectiveness (VE) identified in 17 studies that investigated VE against different endpoints. Pooled VE was 63.1% (95% confidence interval (CI): 40.9–76.9) against asymptomatic infection, 75.7% (95% CI: 69.3–80.8) against symptomatic infection and 90.9% (95% CI: 84.5–94.7) against hospitalisation. Compared with the Alpha variant, VE against mild outcomes was reduced by 10–20%, but fully maintained against severe COVID-19.

The Delta variant (Phylogenetic Assignment of Named Global Outbreak (Pango) lineage designation B.1.617.2) of severe acute respiratory syndrome coronavirus 2 (SARS-CoV-2) was first reported in October 2020 and has spread to more than 180 countries globally [1]. Concerns were raised about how well the currently available vaccines protect against this variant. Since January 2021, the Robert Koch Institute (RKI), in collaboration with the National Immunisation Technical Advisory Groups (NITAGs) network coordinated by the European Centre for Disease Prevention and Control (ECDC) is performing a living systematic review on the efficacy, effectiveness and safety of coronavirus disease (COVID-19) vaccines authorised in the European Union (EU) (PROSPERO registration: CRD42020208935) [2]. Here we present results on the effectiveness and the duration of protection provided by the EU-licensed COVID-19 vaccines in respect to the Delta variant.

## Literature search

This living systematic review follows the Preferred Reporting Items for Systematic Review and Meta-Analysis (PRISMA) guideline (Supplement Part S1). We considered studies of any design as long as they had a comparison group that investigated vaccine effectiveness (VE) against SARS-CoV-2 infection of any severity after vaccination with a COVID-19 vaccine (see Supplement Part S2 for complete population intervention comparison outcomes (PICO) question) approved by the European Medicines Agency. We did not apply any restrictions on publication language and status.

We searched the internal COVID-19 literature database constructed by the RKI library and hand-searched relevant websites (see [2] and Supplement Part S3 for description of the database and the search strategy). Potentially relevant publications were screened at title/abstract and full-text level by at least two out of three independent investigators (TH, WKS, SR). Disagreements on eligibility were resolved through discussion. Data were extracted from the included studies (see PROSPERO protocol for details) and summarised in tables. Only VE estimates of completed vaccination schedules were analysed. The risk of bias in non-randomized studies – of interventions (ROBINS-I) was used to assess risk of bias [3].

We performed meta-analyses, using a random-effects model to account for heterogeneity between studies. The I^2^ was used to quantify the extent of heterogeneity. Formal testing for publication bias was done for datasets with 10 or more estimates by inspection of funnel plots, followed by Begg’s test and Egger’s test (Supplement Part S4).

### Study screening

In total, 7,117 entries were identified and screened until 25 August 2021, the date of last search. Additionally, 11 potentially relevant studies were identified by hand-searching. After full-text screening, 17 studies [4-20] were included (Supplement Part S5: PRISMA flowchart).

### Types of studies

Included studies reported VE against infections with the SARS-CoV-2 Delta variant only, or compared VE against the Delta variant with estimates against the Alpha (B.1.1.7) variant. Within each of these study types, two methodological subtypes were identified: (i) studies that calculated VE against Delta and Alpha from sequenced samples; or (ii) studies that calculated VE from time periods during which Delta and Alpha was the dominant strain in the respective study location without having sequenced each sample. Two studies [7,19] also investigated VE at several time points after vaccination, thereby addressing waning vaccine-induced immunity.

### Prevention of any infection

**Figure 1:**
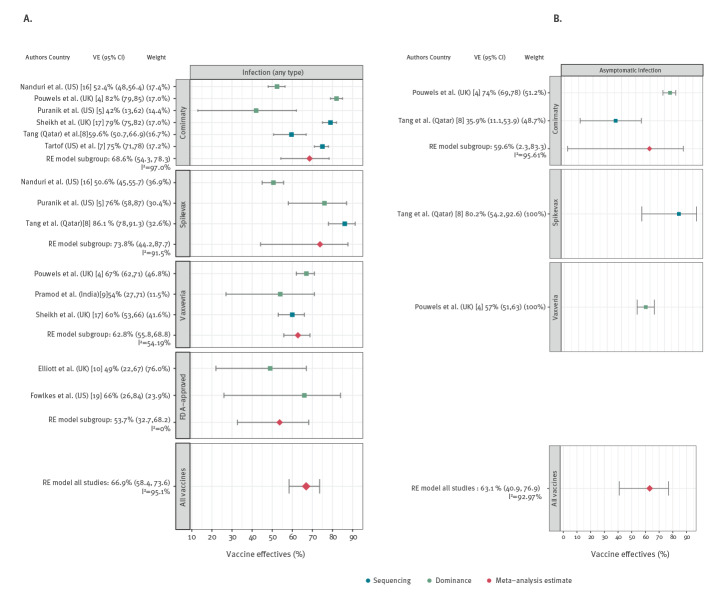
Results of the meta-analysis for SARS-CoV-2 infection outcomes, 1 January–25 August 2021

FDA: The United States Food and Drug Administration; RE: Random effects; SARS-CoV-2: severe acute respiratory syndrome coronavirus 2; UK: United Kingdom; US: United States.

Of 17 studies, 10 [4-10,16,17,19] reported the effectiveness of COVID-19 vaccines in preventing SARS-CoV-2 infection but did not report whether these were symptomatic or asymptomatic infections; Table 1. Studies were conducted in four countries. Five were cohort studies, three were test-negative case–control studies and two were based on serial cross-sectional samples. Six studies investigated more than one vaccine (Comirnaty (BNT162b2 mRNA, BioNTech-Pfizer, Mainz, Germany/New York, United States (US)) and Spikevax (mRNA-1273, Moderna, Cambridge, US) or Comirnaty and Vaxzevria (ChAdOx1 nCoV-19, Oxford-AstraZeneca, Cambridge, United Kingdom (UK) and COVID-19 Vaccine Janssen (Ad26.COV2-S, Janssen-Cilag International NV, Beerse, Belgium). One study each evaluated Comirnaty and Covishield (Vaxzevria, Serum Institute of India, Pune, India) and two studies did not specify the examined vaccines. The VE estimates in the included studies against any type of infection for all age groups, ranged between 49% and 82%; in one study with information for 18–34-year-olds the range was 90% [4]. Pooled VE was 66.9% (95% confidence interval (CI): 58.4–73.6; I^2^ = 95.1%) across all studies (Figure 1 A).

**Table 1:** Effectiveness of COVID-19 vaccines against SARS-CoV-2 infection, 1 January–25 August 2021

Study and publication date	Country	Study design	Study population (n)	Age (years)	Vaccine(s)	Time point of analysis after full vaccination schedule	Adjusted vaccine efficacy/effectiveness (95% CI)			
							Alpha (sequenced)	Delta (sequenced)	Alpha-dominance	Delta-dominance
**Infection (any type)^a^**										
Elliott [10]; 4 August 2021^b^	UK	Serial cross-sectional design	General population (n = 57,457)	18–64	NR	NR	NA	NA	NA	49% (22–67)
Fowlkes [19]; 24 August 2021	US	Cohort study	Frontline workers (HCW and other essential and frontline workers) (n = 4,217), of which 3,483 were vaccinated. (Comirnaty: n = 2,278; Spikevax: n = 1,138; Janssen: n = 67)	≥ 18	Comirnaty; Spikevax; COVID-19 Vaccine Janssen	≥ 14 days	NA	NA	91% (81–96)	66% (26–84)
Nanduri [16]; 18 August 2021	US	Cohort study	Care home residents (Delta-dominance: 5,011,746 vaccinated and 953,861 unvaccinated; Alpha-dominance: 936,123 vaccinated and 217,534 unvaccinated)	Elderly people^c^	Comirnaty; Spikevax	≥ 14 days	NA	NA	Corminaty: 74.2% (68.9–78.7); Spikevax: 74.7% (66.2–81.1)	Comirnaty: 52.4% (48.0–56.4); Spikevax: 50.6% (45.0–55.7)
Pouwels [4]; 24 August 2021^b^	UK	Cohort study	Household members (n = 384,543 for Alpha dominant period; 358,983 during Delta-dominant period)	≥ 18^c^	Comirnaty; Vaxzevria	≥ 14 days	NA	NA	Comirnaty: 78% (68–84); Vaxzevria: 79% (56–90)	Corminaty: 80% (77–83); Vaxzevria: 67% (62–71)
			Household members (n = 358,983)	18–64	Comirnaty; Vaxzevria	≥ 14 days	NA	NA	NA	Comirnaty: all age groups: 82% (79–85) 18–34 years: 90% (85–93%) 35–64 years: 77% (65–85%) Vaxzevria: all age groups: 67% (62–71) 18–34 years: 73% (65–80%) 35–64 years: 54% (40–65%)
Pramod [9]; 22 July 2021^b^	India	Test-negative design	HCW (n = 360 case–control pairs)	Median age: Cases: 34 (28–43), Controls: 33 (28–42)	Covishield	≥ 14 days	NA	NA	NA	54% (27–71)
Puranik [5]; 21 August 2021^b^	US	Cohort study	Vaccinated persons tested for SARS-CoV-2 at Mayo Clinic and affiliated hospitals (Comirnaty: n = 119,463; Spikevax: n = 60,083)	≥ 18^c^	Comirnaty; Spikevax	≥ 14 days	NA	NA	Comirnaty: 76% (69–81); Spikevax: 86% (81–90.6)	Comirnaty: 42% (13–62); Spikevax: 76% (58–87)
Rosenberg [6]; 18 August 2021	US	Serial cross-sectional design	General population (10,135,322 vaccinated and 3,742,197 unvaccinated)	≥ 18^c^	NR	≥ 14 days	NA	NA	91.7%	79.8%
Sheikh [17]; 14 June 2021	UK	Test-negative design	General population (Delta: 53,679 vaccinated with Comirnaty and 32,719 vaccinated with Vaxzevria, 953,861 unvaccinated; Alpha: 53,575 vaccinated with Comirnaty and 32,588 vaccinated with Vaxzevria, 119,419 unvaccinated)	≥ 18^c^	Comirnaty; Vaxzevria	≥ 14 days	Comirnaty: 92% (90–93); Vaxzevria: 73% (66–78)	Comirnaty: 79% (75–82); Vaxzevria: 60% (53–66)	NA	NA
Tang [8]; 11 August 2021^b^	Qatar	Test-negative design	Resident population (Comirnaty: n = 877,354; Spikevax: n = 409,041)	≥ 18^c^	Comirnaty; Spikevax	≥ 14 days	NA	Comirnaty: 59.6% (50.7–66.9) Spikevax: 86.1% (78.0–91.3)	NA	NA
Tartof [7]; 23 August 2021^b^	US	Cohort study	Insurance members (n = 3,436,957)	≥ 12	Comirnaty	≥ 7 days	91% (88‒92)	75% (71‒78)	NA	NA
**Asymptomatic infection**										
Pouwels [4]; 24 August 2021^b^	UK	Cohort study	Household members (n = 358,983)	18–64	Comirnaty; Vaxzevria	≥ 14 days	NA	NA	NA	Comirnaty: 74% (69–78) Vaxzevria: 57% (51–63)
Tang [8]; 11 August 2021^b^	Qatar	Test-negative design	Resident population; (Comirnaty: 877,354; Spikevax: 409,041)	≥ 18^c^	Comirnaty; Spikevax	≥ 14 days	NA	Comirnaty: 35.9% (11.1–53.9) Spikevax: 80.2% (54.2–92.6)	NA	NA

CI: confidence interval; COVID-19: coronavirus disease; HCW: healthcare workers; NA: not applicable; NR: not reported; SARS-CoV-2: severe acute respiratory syndrome coronavirus 2; UK: United Kingdom; US: United States.

^a^ Not reported whether these were symptomatic or asymptomatic infections.

^b^ Preprint.

^c^ Exact age not provided.

Comirnaty (BNT162b2 mRNA, BioNTech-Pfizer, Mainz, Germany/New York, United States (US)), Spikevax (mRNA-1273, Moderna, Cambridge, US), Vaxzevria (ChAdOx1 nCoV-19, Oxford-AstraZeneca, Cambridge, United Kingdom (UK), COVID-19 Vaccine Janssen (Ad26.COV2-S, Janssen-Cilag International NV, Beerse, Belgium). Covishield (Vaxzevria, Serum Institute of India, Pune, India).

The y-axis illustrates study and reported VE estimates (95 % CI) included in the meta-analysis (inverse variance weight for subgroup meta-analysis %). The bottom box represents the results of the meta-analysis across all studies for the outcome (RE model all studies: Pooled VE% (95% CI) I-square statistic for all studies meta-analysis).

FDA-approved: Comirnaty (BNT162b2 mRNA, BioNTech-Pfizer, Mainz, Germany/New York, United States (US)), Spikevax (mRNA-1273, Moderna, Cambridge, US), COVID-19 Vaccine Janssen (Ad26.COV2-S, Janssen-Cilag International NV, Beerse, Belgium).

### Prevention of asymptomatic infection

Two studies investigated VE against asymptomatic infections (Table 1). These studies were performed in the UK and Qatar [4,8]. Both studies investigated two vaccines. The VE estimates ranged between 35.9% and 80.2%. Across studies, pooled VE was 63.1% (95% CI: 40.9–76.9; I^2^ = 93%; Figure 1 B).

### Prevention of symptomatic infection

**Figure 2:**
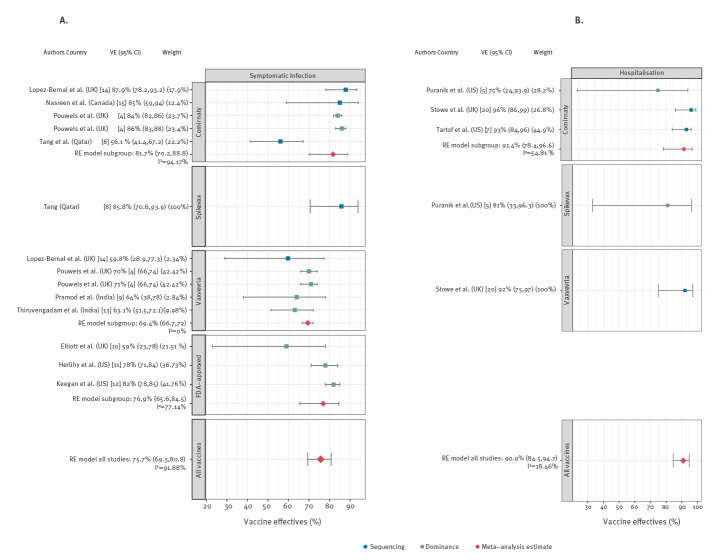
Results of the meta-analyses for symptomatic infection and hospitalisation stratified by vaccine, 1 January–25 August 2021

CI: confidence interval; FDA: The United States Food and Drug Administration; RE: Random effects; SARS-CoV-2: severe acute respiratory syndrome coronavirus 2; UK: United Kingdom; US: United States.

Nine studies estimated the effectiveness of COVID-19 vaccines in preventing symptomatic SARS-CoV-2 infections (Table 2). Studies were performed in five countries [4,8-15]. Five studies had a test-negative design, two studies applied the screening method and one study each had a cohort design and used serial cross-sectional samples. Six studies investigated more than one vaccine. The VE against symptomatic infection ranged between 56% und 87.9%. The pooled VE estimate was 75.7% (95% CI: 69.3–80.8; I^2^ = 91.9%; Figure 2 A).

**Table 2:** Effectiveness of COVID-19 vaccines against SARS-CoV-2 symptomatic infection, severe disease^a^ and hospitalisation, 1 January–25 August 2021

Study and publication date	Country	Study design	Study population (n)	Age (years)	Vaccine(s)	Time point of analysis after full vaccination schedule	Adjusted vaccine efficacy/effectiveness (95% CI)			
							Alpha	Delta	Alpha-dominance	Delta-dominance
**Symptomatic infection**										
Elliott [10]; 4 August 2021^b^	UK	Serial cross-sectional design	General population (n = 57,457)	18–64	NR	NR	NA	NA	NA	59% (23–78)
Herlihy [11]; 6 August 2021	US	Screening method according to Farrington	General population (n = 1,945)	All age groups	Comirnaty; Spikevax; Janssen	≥ 14 days	NA	NA	NA	78% (71–84)
Keegan [12]; 10 August 2021^b^	US	Screening method according to Farrington	Vaccinated people (n = 1,407,233)	All age groups	Comirnaty; Spikevax; Janssen	≥ 14 days	NA	NA	NA	82% (78–85)
Lopez-Bernal [14]; 24 May 2021^b^	UK	Test-negative design	General population (n = 12,675 sequenced cases)	≥ 18^c^	Comirnaty; Vaxzevria	≥ 14 days	Comirnaty: 93,4% (90,4–95,5); Vaxzevria: 66,1% (54–75	Comirnaty: 87,9% (78,2–93,2); Vaxzevria: 59,8% (28,9–77,3)	NA	NA
Nasreen [15]; 16 June 2021^b^	Canada	Test-negative design	General population (n = 421,073)	≥ 16	Comirnaty	≥ 14 days	89% (87–91)	85% (59–94)	NA	NA
Pramod [9]; 22 July 2021^b^	India	Test-negative design	HCW (n = 203 case–control pairs)	Median age: Cases: 34 (28–43), Controls: 33 (28–42)	Covishield (Vaxzevria)	≥ 14 days	NA	NA	NA	64% (38–78)
Pouwels [4]; 24 August 2021^b^	UK	Cohort study	Household members (n = 384,543 for alpha dominant period; 358,983 during delta-dominant period)	≥ 18	Comirnaty; Vaxzevria	≥ 14 days	NA	NA	Comirnaty: 97% (96–98); Vaxzevria: 97% (93–98)	Comirnaty: 84% (82–86); Vaxzevria: 71% (66–74)
			Household members (n = 358,983)	18–64	Comirnaty; Vaxzevria	≥ 14 days	NA	NA	NA	Comirnaty: All age groups: 86% (83–88); 18–34 years: 96% (93–98%) 35–64 years: 88% (78–94%) Vaxzevria: All age groups: 70% (66–74); 18–34 years: 76% (67–83%) 35–64 years: 57% (39–70%)
Tang [8]; 11 August 2021^b^	Qatar	Test-negative design	Resident population; (Comirnaty: 877,354; Spikevax: 409,041)	≥ 18^c^	Comirnaty; Spikevax	≥ 14 days	NA	Comirnaty: 56.1% (41.4–67.2) Spikevax: 85.8% (70.6–93.9)	NA	NA
Thiruvengadam [13]; 16 July 2021^b^	India	Test-negative design	People attending of Employee State Insurance Medical College Hospital or Translational Health Science and Technology Institute, Faridabad for PCR-testing (cases: 2766, controls: 2377)	Median age: cases: 35 (28–45), controls: 32 (26–42)	ChAdOx1 nCOV-19 (Vaxzevria)	≥ 14 days	NA	NA	NA	63.1% (51.5–72.1)
**Severe disease**										
Chia [18]; 31 July 2021^b^	Singapore	Cohort study	Hospitalized patients (n = 218)	≥ 18^c^	mRNA (Comirnaty, Spikevax)	≥ 14 days	NA	93% (66–98)	NA	NA
Tang [8]; 11 August 2021^b^	Qatar	Test-negative design	Resident population; (Comirnaty: n = 877,354; Spikevax: n = 409,041)	≥ 18^c^	Comirnaty; Spikevax	≥ 14 days	NA	Comirnaty: 97.3% (84.4–99.5) Spikevax: 100%^c^	NA	NA
Thiruvengadam [13]; 16 July 2021^b^	India	Test-negative design	People attending of Employee State Insurance Medical College Hospital or Translational Health Science and Technology Institute, Faridabad for PCR-testing (cases: 2766, controls: 2377)	All age groups, median age 35 (cases), 32 (controls)	ChAdOx1 nCOV-19 (Vaxzevria)	≥ 14 days	NA	NA	NA	81.5% (9.9–99.0)
**Hospitalisation**										
Puranik [5]; 21 August 2021^b^	US	Cohort study	Vaccinated persons tested for SARS-CoV-2 at Mayo Clinic and affiliated hospitals (Comirnaty: n = 119,463; Spikevax: n = 60,083)	≥ 18^c^	Comirnaty; Spikevax	≥ 14 days	NA	NA	Comirnaty: 85% (73–93); Spikevax: 91.6% (81–97)	Comirnaty: 75% (24–93.9); Spikevax: 81% (33–96.3)
Rosenberg [6]; 18 August 2021	US	Serial cross-sectional design	General population (10,135,322 vaccinated and 3,742,197 unvaccinated)	≥ 18^c^	NR	≥ 14 days	NA	NA	95.30%	95.30%
Stowe [20]; 14 June 2021^b^	UK	Test-negative design	Symptomatic cases (n = 14,019 of which 166 hospitalized)	≥ 18^c^	Comirnaty; Vaxzevria	≥ 14 days	Comirnaty: 95% (78–99); Vaxzevria: 86% (53–96)	Comirnaty: 96% (86–99); Vaxzevria: 92% (75–97)	NA	NA
Tartof [7]; 23 August 2021^b^	US	Cohort study	Insurance members (3,436,957)	≥ 12	Comirnaty	≥ 7 days	95% (90‒98)	93% (84‒96)	NA	NA

CI: confidence interval; HCW: healthcare workers; NR: not reported.

^a ^The definitions for severe disease are given in Supplement Part S8.

^b^ Preprint.

^c^ Exact age not provided.

Comirnaty (BNT162b2 mRNA, BioNTech-Pfizer, Mainz, Germany/New York, United States (US)), Spikevax (mRNA-1273, Moderna, Cambridge, US), Vaxzevria (ChAdOx1 nCoV-19, Oxford-AstraZeneca, Cambridge, United Kingdom (UK), COVID-19 Vaccine Janssen (Ad26.COV2-S, Janssen-Cilag International NV, Beerse, Belgium). Covishield (Vaxzevria, Serum Institute of India, Pune, India).

The y-axis illustrates study and reported VE estimates (95% CI) included in the meta-analyses (inverse variance weight for subgroup meta-analysis %). The bottom box represents the results of the meta-analysis across all studies for the outcome (RE model all studies: Pooled VE% (95% CI) I-square statistic for all studies meta-analysis).

### Prevention of severe disease and hospitalisation

The VE against the compound outcome severe disease was assessed in three studies (one cohort, two test-negative design). They were performed in three countries (Table 2) [8,13,18]. The VE estimates ranged from 81.5% to 100% and the pooled VE was 93.8% (95% CI: 83–98; I^2^ = 0%; Supplement Part S6). Hospitalisation was reported in four studies: two cohort studies, one test-negative design study and one analysis of cross-sectional serial samples (Table 2). The studies were performed in the US and the UK [5-7,20]. Single study VE ranged between 75% and 96%. Pooled VE against hospitalisation was 90.9% (95% CI: 84.5–94.7; I^2^ = 18.5%; Figure 2 B). No study reported admission to intensive care unit, intubation or death.

### Effectiveness against Delta variant compared with Alpha variant

In nine studies, VE estimates against infections with the Delta variant were compared with those against infections with the Alpha variant in the same study for at least one outcome (Tables 1 and 2). Overall, VE against Delta was 10–20% lower than VE against Alpha for less severe outcomes. For hospitalisation, VE against Delta did not differ from VE against Alpha.

### Waning protection

Two cohort studies from the US investigated VE against infections (symptomatic or asymptomatic) for more than one time point after vaccination. One study reported a decrease of protection offered by the Comirnaty vaccine from 93% (95% CI: 85–97) at baseline to 53% (95% CI: 39‒65) after at least 4 months [7]. The other study investigated protection conferred by any FDA-licensed vaccines in frontline workers (healthcare workers and others) and reported a non-significant change from 85% (95% CI: 68–93) to 73% (95% CI: 49–86) after at least 5 months following full vaccination [19].

### Risk of bias

14 studies [4,5,7-10,13-20] had a moderate risk of bias and three studies [6,11,12] had a critical risk of bias. Major limitations were incomplete or absent adjustment for confounders (see Supplement Part S7 for details). No evidence of publication bias was detected.

## Discussion

These second interim results of our living systematic review show that COVID-19 vaccines approved in the EU have a moderate to high effectiveness against mild to moderate forms of SARS-CoV-2 infections caused by the Delta variant, while VE against severe disease and hospitalisation was high to very high. Statistical heterogeneity was low in meta-analysis of the severe outcomes, further supporting a well-maintained effectiveness against these endpoints under Delta variant dominance.

In one study where VE was investigated in more than one age group, higher estimates in younger groups were seen. Of note, no VE estimate against Delta had been reported until data cut (25 August) for the Janssen vaccine.

As already discussed in the first interim analysis [2] and given the highly dynamic publishing landscape in this field, we cannot exclude the possibility that additional published studies were not captured by our search strategy. Further limitations stem from the fact that genomic sequencing was used to determine VE against Delta in only a minority of studies, while in the majority of studies VE was estimated during time periods of dominant Delta circulation without sequencing. Moreover, based on the current evidence, it is challenging to segregate two factors contributing to the difference between the VE estimates against Alpha and Delta variants: waning immunity and actual VE against Delta. Furthermore, a number of studies did not report separate estimates per vaccine.

### Conclusion

Current evidence shows that COVID-19 vaccines licensed in the EU are moderately to highly effective in preventing SARS-CoV-2 infections with the Delta variant, while effectiveness against severe courses of COVID-19 remains high.

## Supplementary Data

937000 Supplement
