# Supplementary material for: Effectiveness of COVID-19 vaccines against SARS-CoV-2 infection with the Delta (B.1.617.2) variant: second interim results of a living systematic review and meta-analysis, 1 January to 25 August 2021
Source: Euro Surveill. 2021 Oct 14;26(41):2100920. doi: 10.2807/1560-7917.ES.2021.26.41.2100920 (PMC8518304; doi:10.2807/1560-7917.ES.2021.26.41.2100920)
Supplement: Supplement [file 21-00920_HARDER_Supplement.pdf]

Supplementary material

*This supplementary material is hosted by Eurosurveillance as supporting information alongside the article "***Effectiveness of COVID-19 vaccines against SARS-CoV-2 infection with the Delta (B.1.617.2) variant: second interim results of a living systematic review and meta-analysis, 1 January to 25 August 2021***", on behalf of the authors, who remain responsible for the accuracy and appropriateness of the content. The same standards for ethics, copyright, attributions and permissions as for the article apply. Supplements are not edited by Eurosurveillance and the journal is not responsible for the maintenance of any links or email addresses provided therein.*

Contents

S1: PRISMA checklist 2

S2: PICO question 5

S3: Search strategy 6

S4: Meta-analysis: Methods 8

S5: PRISMA-Flowchart 9

S6: Meta-analyses: Results 10

Results: forest plots for infection (any type) 10

Results: forest plots for asymptomatic infection 11

Results: forest plots for symptomatic infection 12

Results: forest plots for hospitalization 13

Results: forest plots for severe disease 13

Results: funnel plots 14

S7: Risk of bias assessments 15

S8: Definitions used by the study authors for the outcome "severe disease" 17

S1: PRISMA checklist

Section/topic	#	Checklist item	Reported on page #
**TITLE**			
Title	1	Identify the report as a systematic review, meta-analysis, or both.	1
**ABSTRACT**			
Structured summary	2	Provide a structured summary including, as applicable: background; objectives; data sources; study eligibility criteria, participants, and interventions; study appraisal and synthesis methods; results; limitations; conclusions and implications of key findings; systematic review registration number.	2
**INTRODUCTION**			
Rationale	3	Describe the rationale for the review in the context of what is already known.	3
Objectives	4	Provide an explicit statement of questions being addressed with reference to participants, interventions, comparisons, outcomes, and study design (PICOS).	3 and Supplement part 2
**METHODS**			
Protocol and registration	5	Indicate if a review protocol exists, if and where it can be accessed (e.g., Web address), and, if available, provide registration information including registration number.	3
Eligibility criteria	6	Specify study characteristics (e.g., PICOS, length of follow-up) and report characteristics (e.g., years considered, language, publication status) used as criteria for eligibility, giving rationale.	3
Information sources	7	Describe all information sources (e.g., databases with dates of coverage, contact with study authors to identify additional studies) in the search and date last searched.	3 and Supplement part 3
Search	8	Present full electronic search strategy for at least one database, including any limits used, such that it could be repeated.	Supplement part 3
Study selection	9	State the process for selecting studies (i.e., screening, eligibility, included in systematic review, and, if applicable, included in the meta-analysis).	3

Data collection process	10	Describe method of data extraction from reports (e.g., piloted forms, independently, in duplicate) and any processes for obtaining and confirming data from investigators.	3-4
Data items	11	List and define all variables for which data were sought (e.g., PICOS, funding sources) and any assumptions and simplifications made.	4
Risk of bias in individual studies	12	Describe methods used for assessing risk of bias of individual studies (including specification of whether this was done at the study or outcome level), and how this information is to be used in any data synthesis.	4
Summary measures	13	State the principal summary measures (e.g., risk ratio, difference in means).	4
Synthesis of results	14	Describe the methods of handling data and combining results of studies, if done, including measures of consistency (e.g., I^2) for each meta-analysis.	4

Section/topic	#	Checklist item	Reported on page #
Risk of bias across studies	15	Specify any assessment of risk of bias that may affect the cumulative evidence (e.g., publication bias, selective reporting within studies).	4
Additional analyses	16	Describe methods of additional analyses (e.g., sensitivity or subgroup analyses, meta-regression), if done, indicating which were pre-specified.	Not applicable
**RESULTS**			
Study selection	17	Give numbers of studies screened, assessed for eligibility, and included in the review, with reasons for exclusions at each stage, ideally with a flow diagram.	4 and Supplement part 5
Study characteristics	18	For each study, present characteristics for which data were extracted (e.g., study size, PICOS, follow-up period) and provide the citations.	4-5, Table 1 and 2
Risk of bias within studies	19	Present data on risk of bias of each study and, if available, any outcome level assessment (see item 12).	Supplement part 5
Results of individual studies	20	For all outcomes considered (benefits or harms), present, for each study: (a) simple summary data for each intervention group (b) effect estimates and confidence intervals, ideally with a forest plot.	Table 1 and 2
Synthesis of results	21	Present results of each meta-analysis done, including confidence intervals and measures of consistency.	4-5, Figure 1 and 2
Risk of bias across studies	22	Present results of any assessment of risk of bias across studies (see Item 15).	Not

			applicable
Additional analysis	23	Give results of additional analyses, if done (e.g., sensitivity or subgroup analyses, meta-regression [see Item 16]).	Not applicable
**DISCUSSION**			
Summary of evidence	24	Summarize the main findings including the strength of evidence for each main outcome; consider their relevance to key groups (e.g., healthcare providers, users, and policy makers).	5
Limitations	25	Discuss limitations at study and outcome level (e.g., risk of bias), and at review-level (e.g., incomplete retrieval of identified research, reporting bias).	5
Conclusions	26	Provide a general interpretation of the results in the context of other evidence, and implications for future research.	5
**FUNDING**			
Funding	27	Describe sources of funding for the systematic review and other support (e.g., supply of data); role of funders for the systematic review.	6

From: Moher D, Liberati A, Tetzlaff J, Altman DG, The PRISMA Group (2009). Preferred Reporting Items for Systematic Reviews and Meta-Analyses: The PRISMA Statement. PLoS Med 6(7): e1000097. doi:10.1371/journal.pmed1000097

S2: PICO question

Participants/population: Male and female participants of all age groups

Intervention: Any vaccine against COVID-19 which has been approved for use in the European Union (or will be approved soon), including complete and incomplete dosing schedules

Comparators/control: placebo, no vaccination or a vaccine not directed against COVID-19 (active comparator), but also including head-to-head trials directly comparing different vaccines against COVID-19

Outcomes: 1. Efficacy and effectiveness-related outcomes: SARS-CoV2 infection (PCR-confirmed); hospitalisation due to COVID-19 (PCR-confirmed); ICU admission due to COVID-19 (PCR-confirmed); intubation and oxygen supply due to COVID-19 (PCR-confirmed); death due to COVID-19 (PCR-confirmed).
2. Safety-related outcomes: local reactions; systemic events; severe adverse events; enhanced COVID-19 disease; adverse events of special interest (AESI), including solicited and unsolicited events

S3: Search strategy

The following searches will be combined with the terms "vaccin*" and "immuniz*" and the brand names of the approved vaccines.*

Search Syntax PubMed 1:
("Severe Acute Respiratory Syndrome Coronavirus 2" [Supplementary Concept] OR "COVID-19" [Supplementary Concept] OR "COVID 19 diagnostic testing" [Supplementary Concept] OR "COVID 19 drug treatment" [Supplementary Concept] OR "COVID 19 serotherapy"[Supplementary Concept] OR "COVID 19 vaccine" [Supplementary Concept] OR "Severe Acute Respiratory Syndrome Coronavirus 2"[tiab] OR ncov*[tiab] OR COVID*[tiab] OR sars-cov-2[tiab] OR "sars cov 2"[tiab] OR "SARS Coronavirus 2"[tiab] OR "Severe Acute Respiratory Syndrome CoV 2"[tiab] OR "Wuhan coronavirus"[tiab] OR "Wuhan seafood market pneumonia virus"[tiab] OR "SARS2"[tiab] OR "2019-nCoV"[tiab] OR "hcov-19"[tiab] OR „novel 2019 coronavirus“[tiab] OR "2019 novel coronavirus*"[tiab] OR „novel coronavirus 2019*“[tiab] OR "2019 novel human coronavirus*"[tiab] OR „human coronavirus 2019“[tiab] OR "coronavirus disease-19"[tiab] OR "corona virus disease-19"[tiab] OR "coronavirus disease 2019"[tiab] OR "corona virus disease 2019"[tiab] OR "2019 coronavirus disease"[tiab] OR "2019 corona virus disease"[tiab] OR „novel coronavirus disease 2019“[tiab] OR „novel coronavirus infection 2019“[tiab] OR "new coronavirus*"[tiab] OR "coronavirus outbreak"[tiab] OR "coronavirus epidemic"[tiab] OR "coronavirus pandemic"[tiab] OR "pandemic of coronavirus"[tiab]) AND ("2019/12/01"[PDAT] : "2099/12/31"[PDAT])

Search Syntax PubMed 2:
("wuhan"[tiab] or china[tiab] or hubei[tiab]) AND ("Severe Acute Respiratory Syndrome Coronavirus 2"[Supplementary Concept] OR "COVID-19" [Supplementary Concept] OR "COVID 19 diagnostic testing"[Supplementary Concept] OR "COVID 19 drug treatment"[Supplementary Concept] OR "COVID 19 serotherapy"[Supplementary Concept] OR "COVID 19 vaccine"[Supplementary Concept] OR "coronavirus*"[tiab] OR "corona virus*"[tiab] OR ncov[tiab] OR COVID*[tiab] OR sars*[tiab])

Search Syntax Embase 1:
('severe acute respiratory syndrome coronavirus 2':ti,ab OR 'severe acute respiratory syndrome coronavirus 2'/exp OR 'COVID 19'/exp OR ncov*:ti,ab OR COVID*:ti,ab OR 'sars cov 2':ti,ab OR 'sars-cov-2':ti,ab OR 'sars coronavirus 2':ti,ab OR 'sars coronavirus 2'/exp OR 'severe acute respiratory syndrome cov 2':ti,ab OR 'wuhan coronavirus':ti,ab OR 'wuhan seafood market pneumonia virus':ti,ab OR sars2:ti,ab OR '2019-ncov':ti,ab OR 'hcov-19':ti,ab OR 'novel 2019 coronavirus':ti,ab OR '2019 novel coronavirus*':ti,ab OR 'novel coronavirus 2019'/exp OR '2019 novel human coronavirus*':ti,ab OR 'human coronavirus 2019':ti,ab OR 'coronavirus disease-19':ti,ab OR 'corona virus disease-19':ti,ab OR 'coronavirus disease 2019':ti,ab OR 'coronavirus disease 2019'/exp OR 'corona virus disease 2019':ti,ab OR '2019 coronavirus disease':ti,ab OR 'novel coronavirus 2019*':ti,ab OR 'novel coronavirus disease 2019':ti,ab OR 'novel coronavirus infection 2019':ti,ab OR '2019 corona virus disease':ti,ab OR 'new coronavirus*':ti,ab OR 'coronavirus outbreak':ti,ab OR 'coronavirus epidemic':ti,ab OR 'coronavirus pandemic':ti,ab OR 'pandemic of coronavirus':ti,ab OR 'severe acute respiratory syndrome coronavirus 2 vaccine'/exp OR 'COVID 19 vaccine'/exp) AND 2020:py

Search Syntax Embase 2:
(wuhan:ti,ab OR china:ti,ab OR hubei:ti,ab) AND ('severe acute respiratory syndrome coronavirus 2':ti,ab OR 'severe acute respiratory syndrome coronavirus 2'/exp OR 'severe acute respiratory

syndrome coronavirus 2' OR 'COVID*':ti,ab OR 'COVID 19'/exp OR 'COVID 19' OR coronavirus*:ti,ab OR 'corona virus*':ti,ab OR ncov:ti,ab OR COVID*:ti,ab OR sars*:ti,ab OR 'sars coronavirus 2'/exp)

Manual search in ArRvix, BioRvix, ChemRvix, MedRvix, Preprints.org, ResearchSquare und SSRN

Manual search at Websites of European Centre for Disease Prevention and Control (ECDC), US Centers for Disease Control, Public Health Agency of Canada, Public Health England, Hauté Autorite´de Santé (France) and World Health Organization.

* For the interim analysis on effectiveness against infection with the Delta variant, the following terms were used in addition: variant*; Delta; delta; VOC*; B.1.617*

S4: Meta-analysis: Methods

Individual vaccine effectiveness estimates and confidence intervals reported in the studies were used to produce the pooled estimates. For the meta-analyses, risk ratios and their 95% confidence intervals were calculated as RR = (1-VE)/100%. Random-effects models with inverse variance weighting were applied for meta-analyses using R, *metafor* package. The meta-analyses were conducted stratifying the estimates by outcomes (infection (any), asymptomatic infection, symptomatic infection, hospitalisation, and severe disease).

For each outcome, random effect models were applied for vaccine-stratified subgroups of studies and all studies. The studies which did not report confidence intervals were excluded from the meta-analyses. Heterogeneity between studies was assessed using the I-square statistic. For meta-analyses based on ten or more estimates, the likelihood of publication bias was assessed by examination of funnel plots, followed by Egger's test and Begg's test.

S5: PRISMA-Flowchart

Date of last search: 25 August 2021

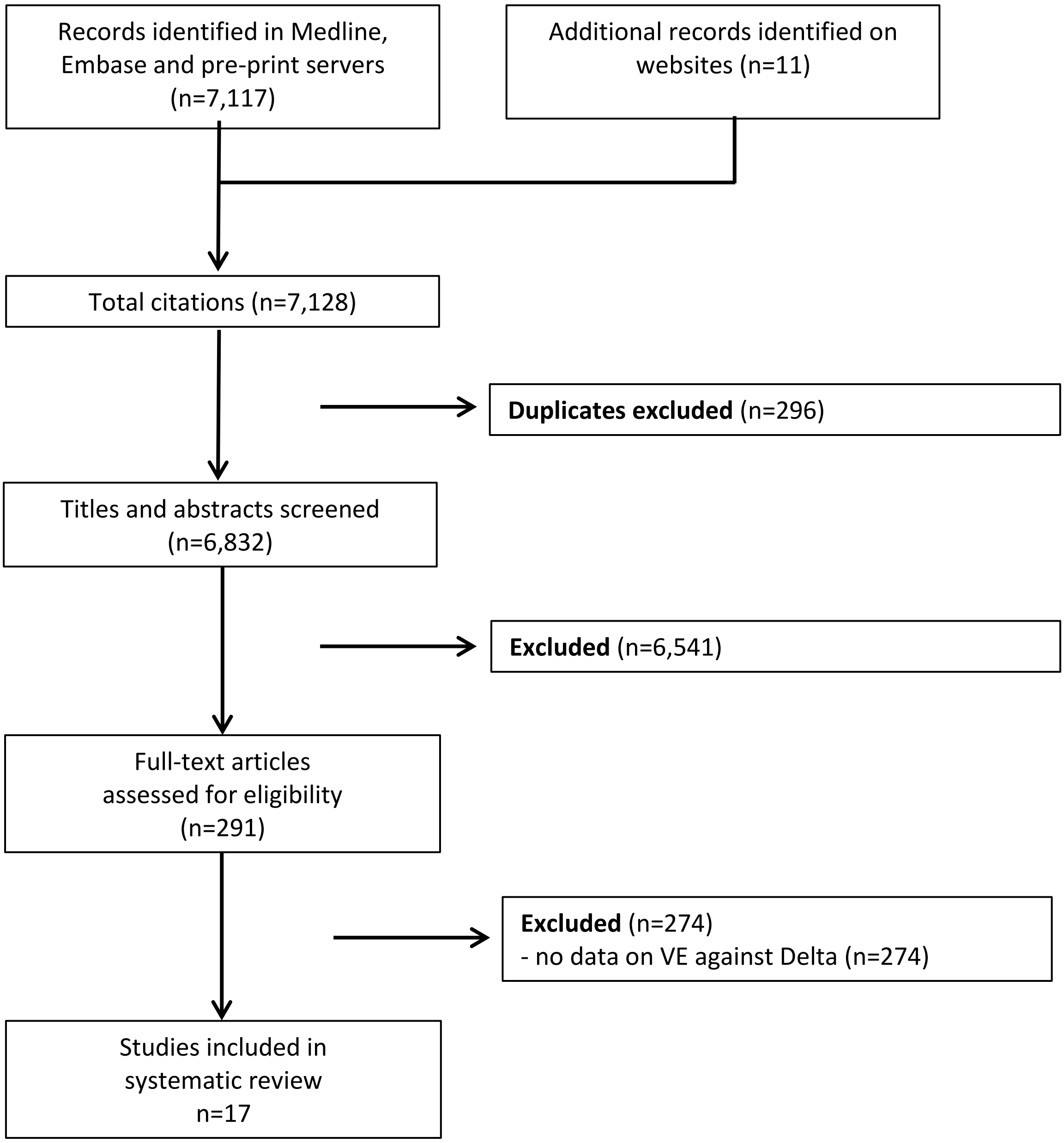

S6: Meta-analyses: Results

Results: forest plots for infection (any type)

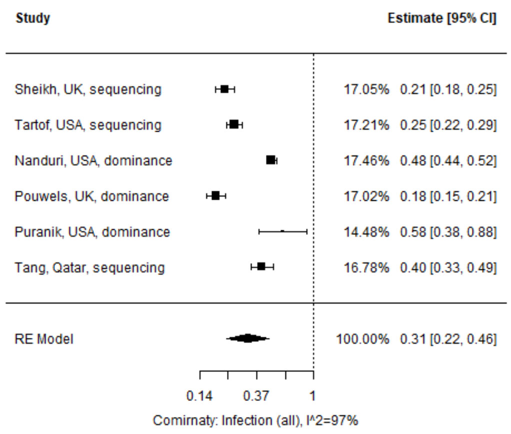

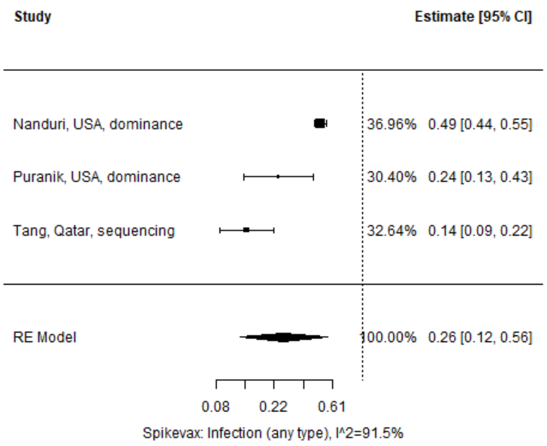

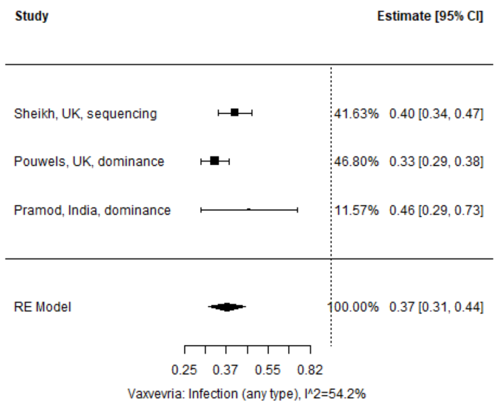

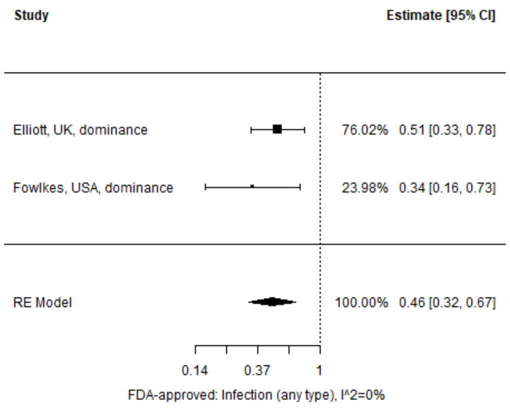

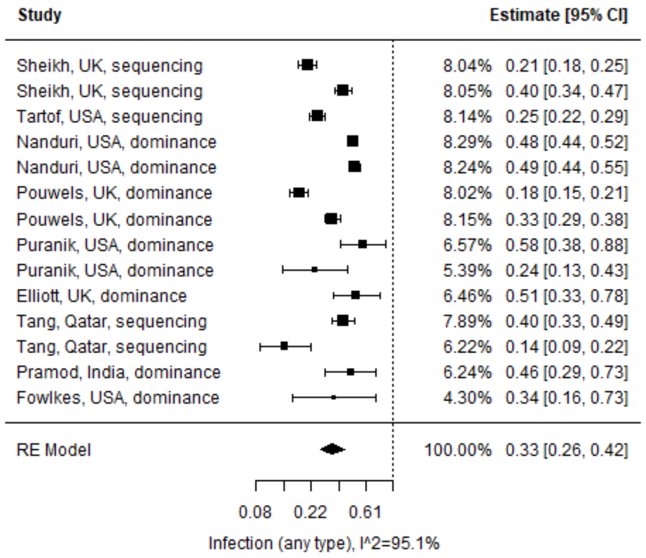

Results: forest plots for asymptomatic infection

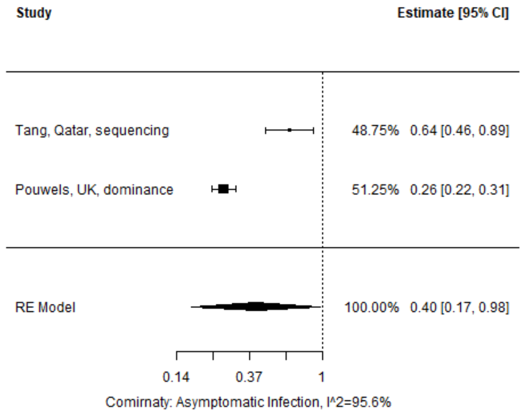

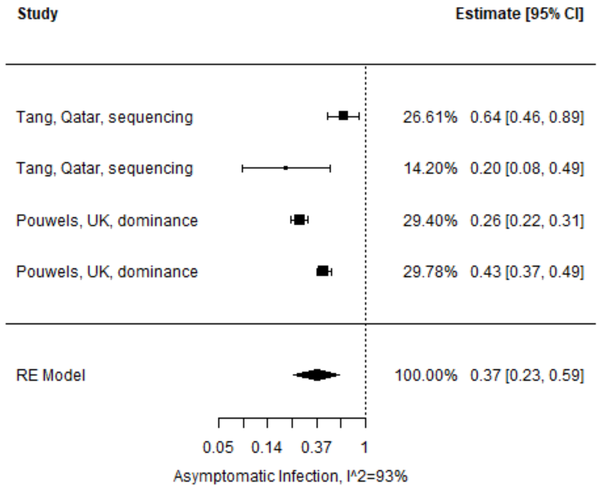

Results: forest plots for symptomatic infection

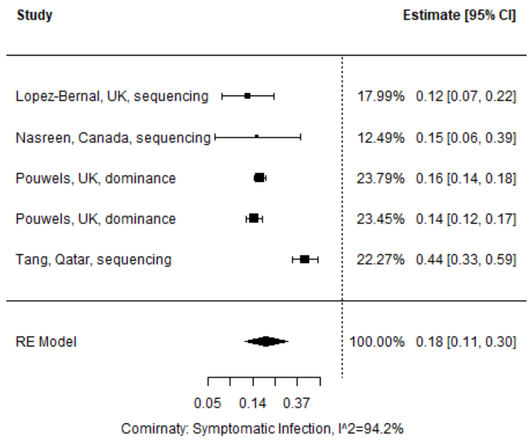

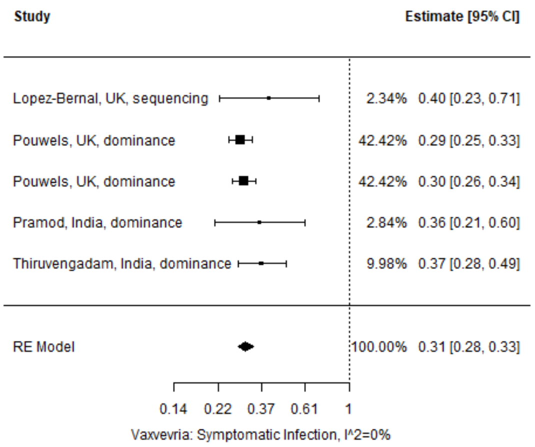

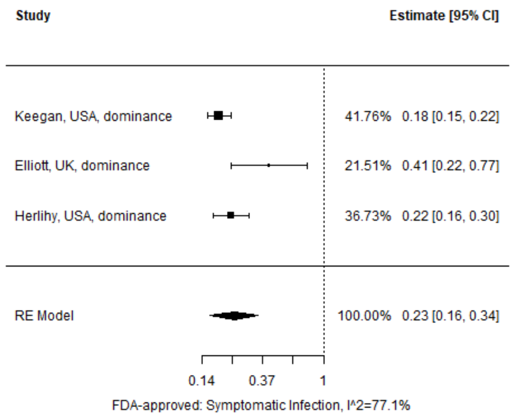

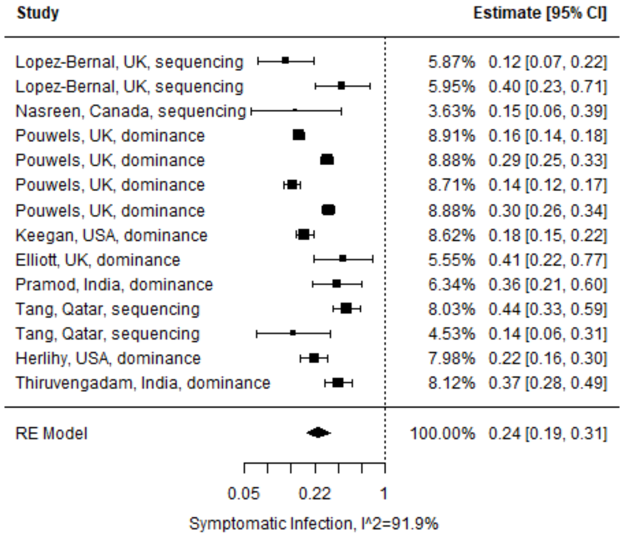

Results: forest plots for hospitalization

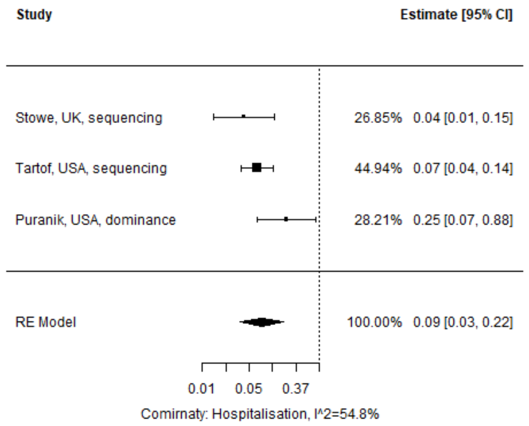

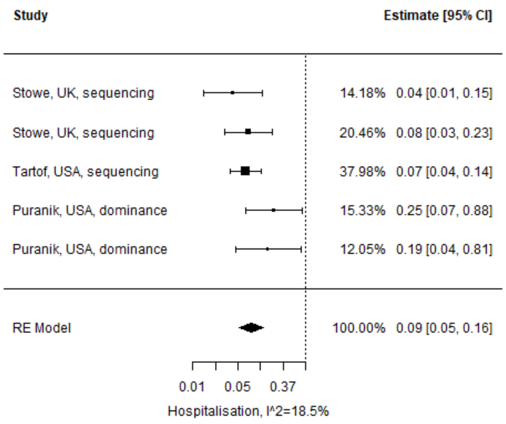

Results: forest plots for severe disease

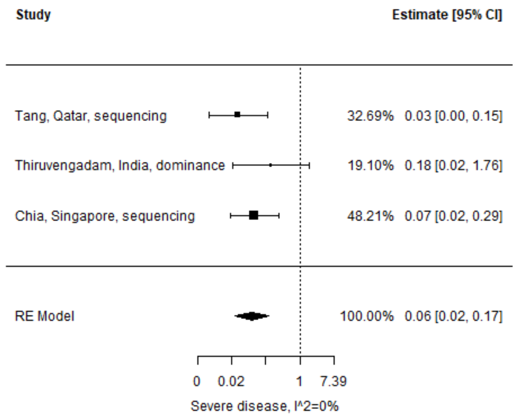

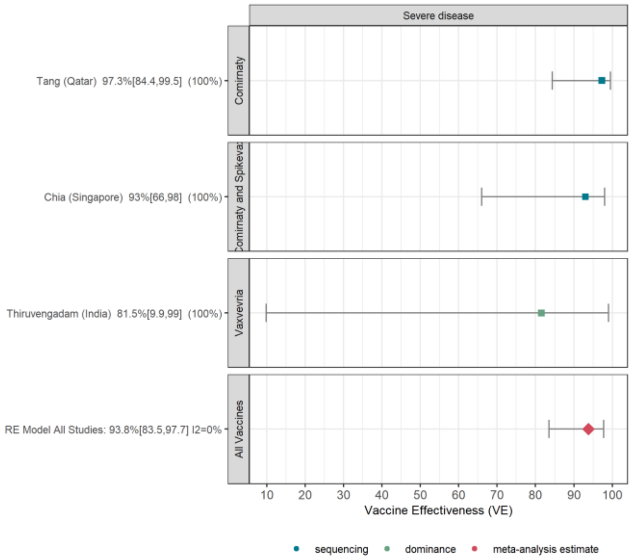

Results: funnel plots

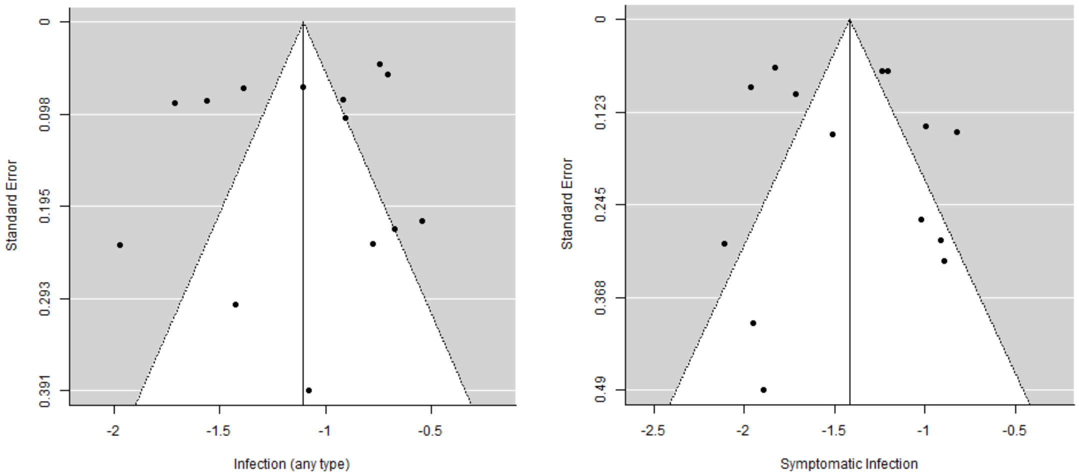

S7: Risk of bias assessments

Study	Bias due to confounding	Bias in selection of participants into the study/analysis	Bias in classification of interventions	Bias due to deviations from intended interventions	Bias due to missing data	Bias in measurement of outcomes	Bias in selection of reported result	Summary
Chia	moderate [1]	low	moderate [5]	low	low	low	low	moderate
Elliott	moderate [1]	low	low	low	low	moderate [2]	low	moderate
Fowlkes	moderate [1]	low	low	low	low	moderate [2]	low	moderate
Herlihy	critical [4]	low	moderate [5]	low	low	moderate [2]	low	critical
Keegan	critical [4]	low	moderate [5]	low	low	moderate [2]	low	critical
Lopez-Bernal	moderate [6]	low	low	low	low	low	low	moderate
Nanduri	moderate [1]	low	low	low	low	moderate [2]	low	moderate
Nasreen	moderate [6]	low	low	low	low	low	low	moderate
Pouwels	moderate [1]	low	moderate [3]	low	low	moderate [2]	low	moderate
Pramod	moderate [6]	low	moderate [3]	low	low	moderate [2]	low	moderate
Puranik	moderate [1]	low	low	low	low	moderate [2]	low	moderate
Rosenberg	critical [4]	low	moderate [5]	low	low	moderate [2]	low	critical
Sheikh	moderate [6]	low	low	low	low	low	low	moderate

Stowe	moderate [6]	low	low	low	low	low	low	moderate
Tang	moderate [6]	low	low	low	low	low	low	moderate
Tartof	moderate [1]	low	low	low	low	low	low	moderate
Thiruvengadam	moderate [6]	low	moderate [3]	low	low	moderate [2]	low	moderate

[1] adjusted estimates reported, but residual confounding possible; [2] VE not based on sequencing of Delta; [3] at least in in some participants, vaccination status was only self-reported; [4] no confounder-adjusted estimates reported; [5] vaccine (product) not reported; [6] test-negative design

S8: Definitions used by the study authors for the outcome "severe disease"

Chia et al.: requiring supplemental oxygen

Tang et al.: SARS-CoV-2 infected person with oxygen saturation of <90% on room air, and/or respiratory rate of >30 breaths/minute, and/or signs of severe respiratory distress (accessory muscle use and inability to complete full sentences)

Thiruvengadam et al.: at least one of the following: the need for oxygen supplementation, admission to intensive care, mechanical ventilation, or death
